# Existence of Similar *Leptospira* Serovars among Dog Keepers and Their Respective Dogs in Mwanza, Tanzania, the Need for a One Health Approach to Control Measures

**DOI:** 10.3390/pathogens10050609

**Published:** 2021-05-16

**Authors:** Betrand Msemwa, Mariam M. Mirambo, Vitus Silago, Juma M. Samson, Khadija S. Majid, Ginethon Mhamphi, Joseph Genchwere, Subira S. Mwakabumbe, Elifuraha B. Mngumi, Georgies Mgode, Stephen E. Mshana

**Affiliations:** 1 Department of Medical Laboratory Sciences, Institute of Allied Health Sciences, Catholic University of Health and Allied Sciences, Bugando, Mwanza P.O. Box 1464, Tanzania; b.msemwa@bugando.ac.tz; 2 Department of Microbiology and Immunology, Catholic University of Health and Allied Sciences, Bugando, Mwanza P.O. Box 1464, Tanzania; vsilago@bugando.ac.tz (V.S.); jumamakoko@bugando.ac.tz (J.M.S.); mshana72@bugando.ac.tz (S.E.M.); 3 Department of Veterinary Medicine and Public Health, Sokoine University of Agriculture, Morogoro P.O. Box 3021, Tanzania; khadijasaid@sua.ac.tz; 4 SUA Pest Management Centre (SPMC), Sokoine University of Agriculture, Morogoro P.O. Box 3110, Tanzania; mhamphi@sua.ac.tz (G.M.); gmgode@sua.ac.tz (G.M.); 5 Tanzania Veterinary Laboratory Agency (TVLA), Lake Zone, Ministry of Livestock and Fisheries, Mwanza P.O. Box 129, Tanzania; tvla.mwanza@tvla.go.tz; 6 Veterinary Investigation Centre (VIC), Lake Zone, Ministry of Livestock and Fisheries, Mwanza P.O. Box 129, Tanzania; subira.samwel@mifugo.go.tz; 7 Department of Veterinary Anatomy and Pathology, School of Biomedical Sciences, Sokoine University of Agriculture, Morogoro P.O. Box 3016, Tanzania; e01elly@sua.ac.tz

**Keywords:** Mwanza, *Leptospira* antibodies, microscopic agglutination (MAT), serovar Sokoine, zoonotic disease

## Abstract

This study investigated seroepidemiology of *Leptospira* serovars among the dog keepers and their dogs in the city of Mwanza, Tanzania. A total of 205 dog keepers and 414 dogs were tested for *Leptospira* antibodies using a microscopic agglutination test (MAT). The median age of the dog keepers was 26 (inter quartile range (IQR): 17–40) years and median duration of keeping dogs was 36 (IQR: 24–120) months. The seropositivity of *Leptospira* antibodies was (33/205 (16.1%, 95% CI: 11.0–21.1) among dog keepers and (66/414 (15.9%, 95% CI: 12.4–19.4) among dogs, *p* = 0.4745. Among the serovars tested (Sokoine, Grippotyphosa, Kenya, Pomona and Hebdomadis), the most prevalent serovar was Sokoine in both dog keepers and their dogs (93.9% (31/33) vs. and 65.1% (43/66), *p* = 0.009). Thirty-one out of thirty-three seropositive dog keepers (93.9%) had dogs positive for *Leptospira* antibodies with 28 (84.9%) having similar serovars with their respective seropositive dogs. Having tertiary education (AOR: 0.24, 95% CI: 0.07–0.84, *p* = 0.026) independently protected individuals from being *Leptospira* seropositive. More than three quarters of dog keepers had similar serovars as their dogs, necessitating one health approach to control measures in endemic areas.

## 1. Background

Leptospirosis is a neglected zoonotic disease of public health importance affecting different populations across the globe [1,2]. The disease is caused by different serovars of *Leptospira* spp. belong to different serogroups. It should be noted that serovar is the basic taxonomic unit and antigenically similar serovars are grouped in a similar serogroup [3]. Leptospirosis causes direct economic impact to humans such as loss of productivity due to illness, suffering and increased healthcare costs for both humans and animals; thus it contributes to poverty in the affected communities.

Humans are susceptible to infection with a variety of *Leptospira* serogroups; however, certain serogroups show some degree of host specificity, for instance: serogroup Icterohaemorrhagiae mostly infect rats and humans, serogroup Sejroe (serovar Hardjo) commonly infects cattle, serogroup Canicola commonly infects dogs and serogroup Pomona mostly infects pigs [4]. Leptospirosis occurs mostly in rural areas due to inadequate sanitation and poor housing. These factors have been found to increase the risk of exposure to animal reservoirs, especially rodents [4,5].

Rodents are the major reservoirs of leptospirosis, they maintain the infection in nature and serve as sources of infection to humans and animals [5]. These spirochetes reside in the kidney of infected rodents and other reservoir hosts for long periods and they are shed to the environment during urination. The seropositivity of *Leptospira* in dogs has been found to range from 7% in Sao Paulo to 73.2% in Caribbean Island [6,7,8,9] with cut points varying from 1:100 to 1:1000. Dogs have been implicated in causing human transmission in some settings [10]. Humans can acquire infection through contact with urine from infected dogs, though no evidence of zoonotic infection was observed during canine leptospirosis outbreak [11]. 

According to the World Health Organization (WHO) Leptospirosis Burden Epidemiology References Group (LERG), the incidence of leptospirosis is 1.03 cases per 100,000 in the population worldwide. The annual morbidity and mortality caused by leptospirosis worldwide is reported to be 14.7 cases per 100,000 population [12]. Worldwide, the Oceania region has the highest burden of leptospirosis (150.6 cases/100,000 population), followed by South East Asia (55.5), Caribbean (50.6) and East Sub-Saharan Africa (25.6) [2,12]. 

In Tanzania, the annual leptospirosis incidence has been reported to range from 75 to 102 cases per 100,000 population [13]. In a recent study in Tanzania [14], serovars reported in humans were Lora, Sokoine, Hebdomadis and Pomona, while in reservoirs (rodents and shrews) serovars Sokoine and Grippotyphosa were detected using a cut point of 1:160. In Mwanza, Tanzania, serovar Sokoine was detected in 7 of 146 abattoir workers and 11 of 104 meat sellers [15] using a cut point of 1:80. Among dogs, a study conducted in 2018 in Morogoro, observed Sokoine, Pomona, Lora and Grippotyphosa to be common serovars among 232 healthy dogs tested using a cut point of 1:160 [7]. 

Dogs are common companion animals in farming and livestock keeping communities in many tropical regions that carry a high risk of transmission of zoonotic diseases to the owners. Despite high seropositivity of *Leptospira* among the animal population in Tanzania [15,16,17], there is scarcity of information on the seropositivity of *Leptospira* antibodies among dog keepers and their dogs. The current study presents the seropositivity of *Leptospira* serovars among dog keepers and their dogs in Mwanza, Tanzania, in an attempt to improve understanding of leptospirosis in high-risk groups. Such information can be useful in devising control strategies. 

## 2. Materials and Methods

### 2.1. Study Design, Study Area and Study Population

A community based cross sectional study involving dog keepers and their dogs was carried out from May to July 2018 in the city of Mwanza. Mwanza city is situated in northwestern Tanzania on the shores of the Lake Victoria. It is located at latitude 2°31′00″ south and longitude 32°53′59″ east at an elevation of 1144 m above the sea level. The city is divided into two districts: Nyamagana and Ilemela districts, with populations of 363,452 and 343,001, respectively, according to the 2012 census [18]. According to the recent livestock census conducted in the city of Mwanza in 2017, the number of dogs in urban and peri-urban areas of the city was approximately 20,000 dogs.

### 2.2. Sampling Method and Specimens Collection

Sample size was estimated by the Kish Leslie formula (1965) using a prevalence of 15.79% at 95% confidence interval and 5% precision [19], the minimum sample size obtained was 204. All dogs owned by a dog keeper were included in the study. A convenient sampling technique was used to recruit the study participants, whereby participants and their dogs were enrolled as they visit dipping areas until the sample size was reached. A structured data collection tool was used to collect all sociodemographic characteristics and relevant characteristics that are known to be risk factors of Leptospirosis [20,21]. These included: sex (dog keeper), age (dog keeper), residence, marital status, education, animal vaccination status, duration of keeping dogs, hygiene practices after handling dog(s), urine contacts, working in farms, having rodents at home, paddy cultivation, fallow land near home, etc. (Table S1). 

Each owner was given a unique study identification number and their respective dog(s) was given the same number with a letter for example 11 for the owner and 11A, 11B, etc. for the respective dog(s). Qualified veterinary laboratory and human laboratory scientists collected a 5 ml venous blood sample from dogs and dog owners, respectively, and placed them in plain vacutainer tubes. Human samples were transported to the Catholic University of Health and Allied Sciences (CUHAS)-Bugando microbiology laboratory while dog samples were transported to the Tanzania Veterinary Laboratory Agency (TVLA), Mwanza, for processing. In both cases, sera were obtained from whole blood by centrifugation at 2000 rpm for 10 minutes and stored at –40 °C in sterile cryovials prior to laboratory analysis to determine *Leptospira* antibodies. All sera were then transported to the Sokoine University of Agriculture, Pest Management Centre (SPMC), where detection of *Leptospira* antibodies was done by using microscopic agglutination test (MAT) as previously described [22].

### 2.3. Microscopic Agglutination Test for Determination of Leptospiral Antibodies

The microscopic agglutination test (MAT) was performed according to Cole et al. and, Goris and Hartskeerl [22]. Briefly, five *Leptospira* serovars were selected from a list of 10 *Leptospira* serovars recommended for diagnosis of leptospirosis in Africa, namely: *L. kirschneri* serovar Sokoine, *L. kirschneri* serovar Grippotyphosa, *L. interrogans* serovar Pomona, *L. interrogans* serovar Hebdomadis and *L. borgpetersenii* serovar Kenya [23] (Table 1). Selected serovars were grown into *Leptospira* EMHJ medium containing 5-Fluorouracil as a selective inhibitor. The cultures were incubated for 4–7 days until a density of 3 × 10^8^ leptospires/ml was reached. Serum samples were serially diluted from 1:10 to 1:80 and 50µl of live antigen was added to double the dilution to 1:20 to 1:160. The mixture was incubated at 30 °C for 2 h and was examined for agglutination under dark field microscopy. Samples reacting with titer of 1:20 and above were titrated further to determine the antibody levels and set a cut point of ≥1:160 as positive [24] (Table S2).

### 2.4. Data Analysis and Management

Data collected was entered into a Microsoft Excel 2007 sheet and then analyzed using STATA version 12 software. Categorical variables were summarized as proportions with a majority having a response of “YES” or “NO” while continuous variables were summarized as median with interquartile range. Univariate analysis and multivariate logistic regression models were fitted to determine the predictors of *Leptospira* seropositivity among dog keepers. All collected factors were subjected to univariate regression analysis, variables with *p* value of less than 0.2 upon univariate analysis were fitted into the multivariate logistic regression model adjusted by age to establish an adjusted odds ratio and their 95% confidence intervals. The fitness of the model was tested using the Wald test. Variables with *p*-value of less than 0.05 were considered statistically significant.

## 3. Results

### 3.1. Sociodemographic Characteristics

This study included 205 dog keepers and 414 dogs residing in rural or urban areas of Mwanza city, Tanzania. The median age of dog keepers was 26 (inter quartile range (IQR):17–40) years with the majority of them (82.9%) being male. The median duration of keeping dogs was 36 (IQR: 24–120) months. Half of the participants 103 (50.2%) were from urban areas and about half of participants 106 (51.7%) were unmarried. Regarding education level, the slightly majority 133 (64.9%) attained primary education. Most of them, 178 (86.8%), reported having rodents at home (Table 2). The ratio of dogs to dog keepers was 2:1 with median of 1, interquartile range (IQR: 1–2). 

### 3.2. Seropositivity of Leptospira spp. Antibodies among Dog Keepers and Their Respective Dogs

The overall seropositivity of *Leptospira* antibodies among dog keepers was found to be 33/205 (16.1%, 95%, CI: 11.1–21.1). Among the five serovars tested in dog keepers (Table 1) the most prevalent was serovar Sokoine, which was detected in 31/33 (93.9 %) of the seropositive individuals while serovar Grippotyphosa was detected in only 3/33 (9.1%) seropositive individuals. Only one participant was seropositive for both serovar Sokoine and Grippotyphosa. 

Among the 414 dogs tested for five serovars (*Leptospira* serovars Sokoine, Grippotyphosa, Kenya, Hebdomadis and Pomona) 66 (15.9%, 95% CI: 12.4–19.4) were found to be seropositive for *Leptospira* spp. antibodies. Serovar Sokoine 43/66 (65.1%) was predominant serovar in dogs followed by serovar Pomona 24/66(36.3%). Serovar Kenya which was not detected in dog keepers contributed 10.6% (7/66) of seropositive dogs (Table 3).

By Wilcoxon (Mann–Whitney) rank sum test, there was no significant difference in duration of keeping dogs among those who were *Leptospira* seropositive and their counterparts (72, IQR: 24–120 vs. 36, IQR: 17–120 months, *p* = 146). No significant difference was observed regarding seropositivity of *Leptospira* antibodies among dog keepers and their respective dogs (Table 2).

### 3.3. Comparison of Leptospira Serovars between Dog Keepers and Their Respective Dogs 

**Figure 1:**
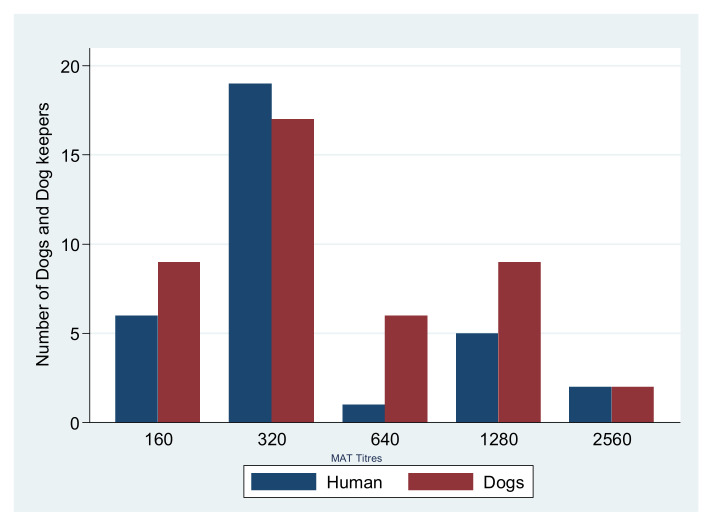
MAT titers for Serovar Sokoine in Dogs and Dog keepers.

Thirty-one out of thirty-three seropositive dog keepers (93.9%) had their dogs tested positive for *Leptospira* antibodies implying that only two seropositive dog keepers had seronegative dogs. Twenty-eight of thirty-three (85%) dog keepers were serovar Sokoine positive (1:160–1:2560), similar to their respective dogs with the same titter range. Figure 1 shows the serovar Sokoine titters among dog keepers and their dogs. 

### 3.4. Factors Associated with Leptospira spp. Seropositivity among Dog Keepers in Mwanza City

On univariate analysis, having a tertiary education (OR 0.23, 95%, CI 0.07–0.80, *p* = 0.021) significantly protected dog keepers from being seropositive. On multivariate logistic analysis having tertiary education remained statistically significantly associated with *Leptospira* spp. seropositivity (OR 0.25, 95% CI 0.07–0.84, *p* = 0.026) (Table 4).

## 4. Discussion

This is the first study to assess the seropositivity of *Leptospira* spp. antibodies among dog keepers and their respective dogs in the city of Mwanza, Tanzania. Seropositivity of *Leptospira* antibodies among dog keepers and dogs were found to be similar in this study suggesting that leptospirosis is endemic in this area both in human and animals. Furthermore, we observed almost all seropositive dog keepers had their respective dogs test positive with similar serovars, suggesting potential transmission of *Leptospira* serovars between humans and dogs. However, due to the observation that 87% of households had rodents, there is possibility that both human and dogs were infected from the same contaminated environment. This underscores the need for a one health approach in tackling pathogens of public health importance and further research using one health approach to address this problem. 

The seropositivity of *Leptospira* antibodies among dog keepers in this study was 16.1%, which is comparable to 12.1% reported in dog owners in Thailand [25] suggesting a similar extent of interactions between dog and dog keepers in these countries. The seropositivity among dog keepers was significantly higher than the 4.5% and 7.8% reported in developed countries [26,27,28,29,30]. This could be explained by the fact that in developed countries dogs’ vaccination against leptospirosis is mandatory by law. In comparison to a previous report in Caribbean Island of Saint Kitts, which reported a seropositivity of 73.2%, the seropositivity reported in the current study is significantly low [6]. The differences in seropositivity could be attributed to differences in seroepidemiology of leptospirosis in these countries. 

In comparison to a recent study among abattoir workers in the same settings, which reported seropositivity of 10.0% [15], the reported seropositivity in this study is significantly high. This could be explained by differences in population whereby exposure to the risk factors might be different among the two populations as evidenced by the fact that dog keepers are at higher risk than abattoir workers [23].

In this study, seropositivity of *Leptospira* antibodies was similar among dog keepers and their dogs whereby serovar Sokoine was found to be predominant serovar in both groups. This observation is similar to the study done in Morogoro, Tanzania, which observed serovar Sokoine to be predominant [7,31] and different from a previous study in Kenya among pigs slaughters that reported *L. interrogans* serovar Lora and *L. borgpetersenii* serovar Kenya to be predominant [32]. The predominance of serovar Sokoine in this study is in agreement with previous findings in the same settings among the abattoir workers suggesting that serovar Sokoine is the most common circulating serovar in different human populations and animals in the city of Mwanza. Studies conducted in other regions of Tanzania also reported *Leptospira* serovar Sokoine as the most prevalent in a broad range of animal hosts and humans [23]. Further studies including isolation of leptospires to explore other possible serovars circulating in Mwanza in different populations are warranted, especially since only five *Leptospira* serovars were used in the MAT out of the 10 *Leptospira* serovars recommended for inclusion in the serological diagnosis of leptospirosis in Africa [23]. Furthermore, we observed serovar Sokoine to be significantly higher in humans than in their respective dogs. Serovar Kenya was detected more in dogs than in dog keepers, suggesting serovars variations among different animal hosts [23].

Among the factors assessed in the current study, having a tertiary education significantly protected dog keepers from being *Leptospira* seropositive. The possible explanation could be due to the fact that, having high education level might be associated with basic knowledge and awareness of diseases and risk factors, particularly animal associated diseases. This emphasizes the need of educating the dog keepers on the risk behaviors that might be associated with acquisition of *Leptospira* infections. Furthermore, there is a need for more research to investigate interactions between dog keepers and dogs and various ways of keeping dogs among people with different levels of education.

### Study Limitation

Vaccinated dogs and unvaccinated dogs often have different positive rates. Additionally, free and enclosed dogs might have different exposure and transmission risks. Some of this information was not captured in this study and due to a small number of vaccinated dogs included, the difference observed was not statistically significant. Five *Leptospira* serovars were used in the MAT out of the 10 *Leptospira* serovars recommended for inclusion in the serological diagnosis of leptospirosis in Africa. This might underestimate the seropositivity; however, the five serovars used formed the majority of cases in previous studies that used 10 serovars. Due to the fact that this study did not include control groups, the data should be carefully interpreted because dog keepers and their respective dog could get similar serovars from an environment that has been contaminated by rodents. 

## 5. Conclusions and Recommendations 

The presence of *Leptospira* antibodies among dog keepers in Mwanza with a high serovars similarity with that of their dogs suggests a potential high risk of transmission of this zoonotic disease. This should give clinicians a high index of suspicion of leptospirosis tin this particular population when they present with fevers of unknown origin. There is a need of advocating for one health approach in tackling zoonotic public health pathogens and considering enforcing vaccination programs among dogs. Moreover, further studies to explore other possible serovars circulating in Mwanza in different populations are warranted. In addition, there is need for a study that will include a control group and predetermined sample size of vaccinated and un-vaccinated dogs. 

## Figures and Tables

**Table 1:** Species, serogroups, serovars and strains used in MAT for the dog keepers and their dogs in Mwanza, Tanzania.

Serial Number	Species	Serogroups	Serovars	Strains
1	*L. kirschneri*	Icterohaemorrhagiae	Sokoine	RMI-Cattle
2	*L. kirschneri*	Grippotyphosa	Grippotyphosa	Moskva-V
3	*L.interrogans*	Pomona	Pomona	Pomona
4	*L.interrogans*	Hebdomadis	Hebdomadis	Hebdomadis
5	*L.borgpetersnii*	Ballum	Kenya	Sh9-giant rats

**Table 2:** Sociodemographic characteristics of 205 dog keepers in Mwanza city.

Participants’ Characteristics	Frequency/Median	Percent (%)/IQR
**Age (years)**	26	17–40
**Duration of keeping dogs (months)**	36	24–120
**Sex**		
Male	170	82.9
Female	35	17.1
**Residence**		
Urban	103	50.2
Rural	102	49.9
**Marital status**		
Married	99	48.3
Single	106	51.7
**Education level**		
Primary	133	64.9
Secondary	59	28.8
Tertiary	13	6.3
**Animal vaccination status**		
Yes	18	8.78
No	187	91.2
**Wash hands after attending animal**		
Yes	137	66.8
No	68	33.2
**Contact urine**		
Yes	15	7.3
No	190	92.7
**Working in farms**		
Yes	61	29.8
No	144	70.2
**Having rodents at home**		
Yes	178	86.8
No	27	13.2
**Paddy cultivation**		
Yes	2	1.0
No	203	99.0
**Fallow land near home (within 100 m)**		
Yes	26	12.7
No	179	87.3

**Table 3:** Seropositivity of different *Leptospira* serovars among dogs and their respective dog keepers in Mwanza, Tanzania.

Serovars/Overall	Dogs (N = 414)	Dog Keepers (N = 205)	*p* Value
Overall	66/414 (15.9%)	33/205 (16.1%)	0.475
Sokoine	43/66 (65.1%)	31/33 (94.0%)	0.001
Pomona	24/66 (36.3%)	Not done	N/A
Kenya	7/66 (10.6%)	0/33 (0.0%)	0.026
Grippotyphosa	4/66 (6.0%)	3/33 (9.1%)	0.285
Hebdomadis	0/66 (0.0%)	0/33 (0.0%)	N/A

**Table 4:** Factors associated with *Leptospira* seropositivity among dog keepers in Mwanza city Tanzania.

Variables	Negative	Positive	Univariate Analysis		Multivariate Analysis	
			OR (95%CI)	*p* Value	OR (95%CI)	*p* Value
	Median % (IQR)	Median % (IQR)				
**Age**	26 (17–40)	24 (19–43)	1.01 (0.99–1.04)	0.322	1.0(0.98–1.03)	0.316
**Sex**						
Female	30 (85.7)	5 (14.3)	1			
Male	142 (83.5)	28 (16.5)	1.18 (0.42–3.31)	0.749		
**Marital status**						
Married	84 (84.8)	15 (15.1)	1			
Single	88 (83.0)	18 (16.9)	1.15 (0.54–2.42)	0.722		
**Education level**						
Primary	116 (87.3)	17 (12.8)	1			
Secondary	48 (81.4)	11 (18.6)	0.23 (0.10–0.80)	0.129	0.42 (0.11–1.61)	0.211
Tertiary	8 (83.9)	5 (38.5)	0.37 (0.68–0.80)	0.021	0.25 (0.07–0.84)	**0.026**
**Residence**						
Urban	87 (84.5)	16 (15.5)	1			
Rural	85 (83.3)	17 (16.7)	1.09 (0.52–2.29)	0.825		
**Animal vaccination status**						
Yes	17 (94.4)	1 (5.5)	1			
No	155 (82.9)	32 (17.1)	3.51 (0.45–27.33)	0.231		
**Wash hands**						
Yes	114 (83.1)	23 (16.8)	1	0.703		
No	58 (85.3)	10 (14.7)	0.85 (0.38–1.92)			
**Contact urine**						
Yes	14 (93.3)	1 (6.7)	1			
No	158 (83.2)	32 (16.8)	0.35 (0.45–2.27)	0.322		
**Working in farms**						
No	123 (85.4)	21 (14.6)	1			
Yes	49 (80.3)	12 (19.7)	1.43 (0.66–3.14)	0.366		
**Rodent at home**						
No	150 (84.3)	28 (15.7)	1			
Yes	22 (81.5)	5 (18.5)	1.22 (0.43–3.48)	0.714		
**Paddy cultivation**						
Yes	2 (100.0)	0 (0.0)				
No	170 (83.7)	33 (16.3)				
**Fallow land near home**						
Yes	23 (88.5)	3 (11.5)	1			
No	149 (83.2)	30 (16.8)	0.65 (0.18–2.30)	0.501		

* Wald test, Chi square = 8.71, *p* = 0.4643.

## Data Availability

All data have been included in the manuscript.

## Supplementary Materials

The following are available online at https://www.mdpi.com/article/10.3390/pathogens10050609/s1, Table S1: Data Collection tool, Table S2: MAT results.
