# Supplementary material for: Existence of Similar Leptospira Serovars among Dog Keepers and Their Respective Dogs in Mwanza, Tanzania, the Need for a One Health Approach to Control Measures
Source: Pathogens. 2021 May 16;10(5):609. doi: 10.3390/pathogens10050609 (PMC8156512; doi:10.3390/pathogens10050609)
Supplement: Supplementary file 1 [file pathogens-10-00609-s001.zip › Supplementary File 1_Data collection tool.pdf]

Supplementary File 1: Data collection tool

	**PARTICIPANT'S PARTICULARS**	
NO	**Question**	**Response**
1.	Date	____/____/20____
2.	Study number	___________________
3.	Mobile phone number	LB-________________
4.	Age (owner/attendant)	________________
5.	Sex (owner/attendant)	1. Male<br>2. Female
6.	Residence (mention place) __________________	1. Urban<br>2. Rural<br>3. Periurban
7.	Marital status	1. Married<br>2. Single<br>3. Widowed<br>4. Divorced
8.	Education level	1. No formal education<br>2. Primary<br>3. Secondary<br>4. Tertiary
9.	Occupation	1. Meat seller/vendor<br>2. Abattoir worker<br>3. Dog keeper/ attendant<br>4. Veterinary worker<br>5. Meat inspector<br>6. Employed
	**ASSOCIATED FACTORS**	
10.	For how long have you been keeping the dog(s)?	Years_________or months______
11.	Did your dog(s) receive vaccine against leptospirosis?	1. YES<br>2. NO
12.	Do you keep other animals different from dogs?	1. YES<br>2. NO
13.	If YES mention them	________________________________<br>________________________________<br>________________________________<br>________________________________
14.	Do you normally wash fruit/vegetables before consumption?	1. YES<br>2. NO
15.	Do you have a habit of eating during work/breaks?	1. YES

		2. NO
16.	Do you wash your hands before and after eating?	1. YES<br>2. NO
17.	Do you always wear protective gears when attending the dog(s)?	1. YES<br>2. NO
18.	If YES which one (s)?	1. Coat<br>2. Gloves<br>3. Apron<br>4. Plastic boots<br>5. Cap<br>6. Face mask<br>7. Other (specify)…………………………
19.	How often do you clean your protective gears?	1. Everyday<br>2. Once a week<br>3. Twice a week<br>4. >2 a week
20.	Do you live in a house with sewage system (public service/septic tank)?	1. YES<br>2. NO
21.	Are you engaged in paddy cultivation activities?	1. YES<br>2. NO
22.	Are there any abandoned plots near your house?	1. YES<br>2. NO
23.	Do you have direct contact with blood/organs from the slaughterhouse?	1. YES<br>2. NO
24.	Do you have any contact with dog urine?	1. YES<br>2. NO
25.	Have you been excluded from attending dog(s) when getting wounds/bruisers?	1. YES<br>2. NO
26.	Have you been working in farms previously?	1. YES<br>2. NO
27.	Have you ever helped animal births?	1. YES<br>2. NO
28.	If YES, did you wear any protective gears?	1. YES<br>2. NO
29.	Do you keep clothes clean after attending your dog(s)?	1. YES<br>2. NO
30.	Do you wash hands after animal contact?	1. YES<br>2. NO
31.	Are you working with bare feet?	1. YES<br>2. NO
32.	Have you ever been splashed with infected fluids (blood, urine, etc)?	1. YES<br>2. NO

33.	Are there any rodents at home?	1. YES<br>2. NO
34.	Is there any fallow land near home(within 100m)	1.YES<br>2:NO
